# Systematic identification of non-coding somatic single nucleotide variants associated with altered transcription and DNA methylation in adult and pediatric cancers

**DOI:** 10.1093/narcan/zcab001

**Published:** 2021-02-01

**Authors:** Fengju Chen, Yiqun Zhang, Chad J Creighton

**Affiliations:** Dan L. Duncan Comprehensive Cancer Center, Baylor College of Medicine, Houston, TX 77030, USA; Dan L. Duncan Comprehensive Cancer Center, Baylor College of Medicine, Houston, TX 77030, USA; Dan L. Duncan Comprehensive Cancer Center, Baylor College of Medicine, Houston, TX 77030, USA; Department of Bioinformatics and Computational Biology, The University of Texas MD Anderson Cancer Center, Houston, TX 77030, USA; Department of Medicine, Baylor College of Medicine, Houston, TX 77030, USA; Human Genome Sequencing Center, Baylor College of Medicine, Houston, TX 77030, USA

## Abstract

Whole-genome sequencing combined with transcriptomics can reveal impactful non-coding single nucleotide variants (SNVs) in cancer. Here, we developed an integrative analytical approach that, as a first step, identifies genes altered in expression or DNA methylation in association with nearby somatic SNVs, in contrast to alternative approaches that first identify mutational hotspots. Using genomic datasets from the Pan-Cancer Analysis of Whole Genomes (PCAWG) consortium and the Children's Brain Tumor Tissue Consortium (CBTTC), we identified hundreds of genes and associated CpG islands for which the nearby presence of a non-coding somatic SNV recurrently associated with altered expression or DNA methylation, respectively. Genomic regions upstream or downstream of genes, gene introns and gene untranslated regions were all involved. The PCAWG adult cancer cohort yielded different significant SNV-expression associations from the CBTTC pediatric brain tumor cohort. The SNV-expression associations involved a wide range of cancer types and histologies, as well as potential gain or loss of transcription factor binding sites. Notable genes with SNV-associated increased expression include *TERT*, *COPS3*, *POLE2* and *HDAC2*—involving multiple cancer types—*MYC*, *BCL2*, *PIM1* and *IGLL5*—involving lymphomas—and *CYHR1*—involving pediatric low-grade gliomas. Non-coding somatic SNVs show a major role in shaping the cancer transcriptome, not limited to mutational hotspots.

## INTRODUCTION

The cancer genome is characterized by widespread somatic genomic alterations, which may include single nucleotide variants (SNVs), genomic rearrangements or structural variants (SVs), small insertions or deletions (indels) and copy number alterations (CNAs). Only a small subset of somatic mutations detected for a given cancer genome is thought to play a critical role in the development or progression of the disease, with such mutations termed as ‘drivers’ as opposed to the presumably non-essential ‘passenger’ mutations (1). At the same time, driver discovery is not yet complete (1,2), and recent findings of medium-impact putative passengers may challenge the driver–passenger dichotomy (3). Much of the early efforts of major cancer genomics initiatives, including The Cancer Genome Atlas (TCGA) and the International Cancer Genomics Consortium (ICGC), focused primarily on somatic mutations occurring within the ∼1% of the genome that is protein-coding. As opposed to whole-exome sequencing, whole-genome sequencing (WGS) would involve the entire genome, including both coding and non-coding alterations. Recent efforts by the Pan-Cancer Analysis of Whole Genomes (PCAWG) consortium represented an important milestone in providing curated TCGA and ICGC WGS data on over 2500 cancers and their matching normal tissues across 38 tumor types (1), with a substantial fraction of these cases also having RNA sequencing or DNA methylation data. Independent efforts by the Children's Brain Tumor Tissue Consortium (CBTTC) (4) provide combined WGS and RNA sequencing data on pediatric brain tumors, where PCAWG expression data did not include any pediatric brain tumor cases.

Much work remains to distinguish impactful from not impactful mutations in cancer, including somatic non-coding SNVs. Perhaps the most well-known example of somatic non-coding SNVs having functional impact involves *TERT*, for which two highly recurrent promoter mutations create ETS family binding sites resulting in *TERT* upregulation (5,6). In general, however, somatic non-coding variants tend not to be recurrent among patients, which makes the characterization of their functional impact particularly challenging (7). Analytical methods to define impactful non-coding SNVs include identifying regions that show clustering of SNVs in the genome across samples, or looking within pre-defined genomic regions of interest (known regulatory regions in particular) (2,7). Also, the integration of WGS with transcriptome data can reveal impactful non-coding cancer-associated alterations that DNA-only approaches might have missed (8). Using the above *TERT* promoter SNVs as a template, pre-defined non-coding regions in proximity to each gene, e.g. 1–2 kb upstream, could be systematically examined for the presence of somatic SNVs, and whether these would be associated with altered gene expression. We have previously used a similar approach to associate SV breakpoints with altered expression (9–11). This approach could identify SNV-expression associations even in cases where the SNVs are not recurrent (so long as they fall within the given region). Furthermore, the approach would not be limited to our current understanding of the functional or regulatory role of the non-coding genome, which remains incomplete.

In this present study, we set out to survey genes with their expression potentially impacted by nearby non-coding somatic SNVs. Our study took advantage of the unique resources and opportunities offered by the PCAWG consortium and the CBTTC—which respectively include cancer profiles of gene expression for over 1200 tumors and over 850 tumors, with corresponding SNV data by WGS. The PCAWG and CBTTC cohorts represent adult cancers and pediatric brain tumors, respectively, where the two would represent different biology and genomics. We utilized an analytical approach integrating RNA with SNV data, which approach we adapted from another that we previously used to identify associations of SV breakpoints with gene expression (9–11). Our approach as a first step identifies gene altered in expression in association with nearby SNVs, regardless of whether the SNVs would cluster in a tight pattern. Our approach would be in contrast to analysis approaches that first identify mutational hotspots and then determine which of these may involve the altered expression of nearby genes (2). Also, we adapted our SNV-expression integration approaches to identifying analogous SNV associations with CpG Island (CGI) methylation, based on the subset of cases in the PCAWG cohort with DNA methylation array data. We found widespread associations of somatic non-coding SNVs with altered gene expression in both adult and pediatric cancers, along with SNV associations with altered DNA methylation. Our results include genes not identified in other recent WGS cancer studies.

## MATERIALS AND METHODS

### Patient cohorts

The results here are based upon publicly available data generated by both the PCAWG project (involving data from both TCGA Research Network and the ICGC) and the CBTTC. Tumor molecular profiling data were generated through informed consent as part of the efforts of each respective project and analyzed here per the specified data use guidelines and restrictions. For the PCAWG cohort, combined WGS analysis and RNA-sequencing (RNA-seq) analysis was carried out for 1220 cases in total (9), with WGS coverage at ∼30–60× for tumors. Cases profiled spanned a range of cancer types (bladder, sarcoma, breast, liver-biliary, cervix, leukemia, colorectal, lymphoma, prostate, eosophagus, stomach, central nervous system or ‘cns’, head/neck, kidney, lung, skin, ovary, pancreas, thyroid and uterus), as detailed in Supplementary Data S1. Of the 1220 PCAWG patients, all but 24 were over the age of 17 (8,9). Of the 1220 cases with WGS and RNA-seq, 568 cases were from TCGA that were also uniformly profiled for DNA methylation using Illumina 450K array platform.

For the CBTTC cohort, combined WGS analysis (at 60× coverage) and RNA-seq analysis (at 30× coverage) were carried out for 854 pediatric brain tumor samples, representing 759 patients. Tumor samples in CBTTC spanned at least 33 different tumor types: APTAD, Adenoma; ATRT, Atypical Teratoid Rhabdoid Tumor; CHDM, Chordoma; CNC, Neurocytoma; CPC, Choroid plexus carcinoma; CPP, Choroid plexus papilloma; CRANIO, Craniopharyngioma; DIPG, Diffuse intrinsic pontine glioma; DNT, Dysembryoplastic neuroepithelial tumor (DNET); EPM, Subependymal Giant Cell Astrocytoma (SEGA); EPMT, Ependymoma; ES, Ewing's Sarcoma; GMN, Germinoma; GNBL, Ganglioneuroblastoma; GNG, Ganglioglioma; GNOS, Glial-neuronal tumor not otherwise specified (NOS); HMBL, Hemangioblastoma; LCH, Langerhans cell histiocytosis; MBL, Medulloblastoma; MNG, Meningioma; MPNST, Malignant peripheral nerve sheath tumor; NBL, Neuroblastoma; NFIB, Neurofibroma/Plexiform; ODG, Oligodendroglioma; PBL, Pineoblastoma; PCNSL, Primary CNS lymphoma; PHGG, High-grade glioma/astrocytoma (WHO grade III/IV); PLGG, Low-grade glioma/astrocytoma (WHO grade I/II); PNET, Supratentorial or Spinal cord primitive neuroectodermal; RMS, Rhabdomyosarcoma; SARCNOS, Sarcoma; SCHW, Schwannoma; TT, Teratoma; and Other/unspecified. A subset of CBTTC samples represented multiple samples taken from the same patient, involving 170 tumor samples from 75 patients in total. As indicated in Supplementary Data S1, multiple samples from the same patient may entail samples from multiple initial tumors, or samples taken at different times, e.g. samples taken initially from the initial tumor and later from a progressive or recurrent tumor. As different tumors from the same patient often demonstrate extensive molecular heterogeneity with respect to each other (12), each sample was analyzed independently in the integrative analyses. The situation with CBTTC is different from that involving studies of intratumoral heterogeneity (13), as in CBTTC multiple independent tumors from the same patient are profiled, rather than multiple samples taken from the same initial tumor.

### Somatic single nucleotide variant (SNV) data

For the PCAWG cohort, we used the consensus set of somatic SNV calls as provided by the PCAWG consortium, which applied multiple analytical pipelines applied to SNV calling, with the resulting variants then merged and subjected to a set of filters and other QC steps as described previously (1). PCAWG genomic data were aligned using hg19 genome coordinates. For the CBTTC cohort, CBTTC data included SNV calls by either Strelka2 v2.9.3 or Mutect2 v4.1.10 algorithms. CBTTC genomic data were aligned using hg38 genome coordinates. We used only variant calls that passed quality filters in the analyses. We considered variant calls made by either Strelka2 or Mutect2, as true SNV calls missed by one variant caller would potentially be supplemented by the inclusion of results from the other caller. Besides, the data integration represented another barrier for false positive SNV calls to contribute to the SNV–gene associations. The integration of results between orthogonal data platforms, namely WGS and RNA-seq, was a key aspect of our study, as associations identified must be significant enough to rise above any noise involving the respective data platforms.

### Gene expression data

PCAWG expression calls by RNA-seq were available for 1220 cases (including 442 ICGC cases and 778 TCGA cases), which data involved alignments by both STAR (version 2.4.0i,2-pass) and TopHat2 (version 2.0.12) being used to generate a combined set of calls, which efforts substantially reduced potential batch effects due to the use of different computational pipelines between ICGC and TCGA projects (14). For *TERT* gene in particular, we used the TopHat2 expression calls. *TERT* represented a positive control (5), but while the combined STAR/TopHat2 expression values were significantly associated with TERT mutation by rank-based statistics (9), these values did not lend themselves to linear modeling for some reason, while the TopHat2 expression calls did. We obtained processed RNA-seq data for 854 CBTTC samples from the PedCBioPortal (https://pedcbioportal.org/), which data we quantile normalized before the analyses.

### Copy number alteration (CNA) data

Gene-level copy number calls were based on WGS analysis results, as provided by PCAWG and CBTTC. PCAWG consortium previously generated gene-level copy calls using a consensus of multiple copy callers (15). For the PCAWG cohort, we used copy number calls of five or higher to approximate gene amplification. For the CBTTC cohort, we obtained gene-level CNA calls from the PedCBioPortal (https://pedcbioportal.org/datasets).

We inferred low-level gene gain (approximating three–four copies), high-level gene amplification (approximating five or more copies), low-level copy loss (approximating heterozygous loss), or deep copy loss (approximating gene deletion) using the ‘thresholded’ calls (with values of +1, +2, −1, or −2, respectively) as made available by PedCBioPortal.

### Integrative analyses between SNVs and gene expression

To examine associations between gene expression and nearby SNVs, we adapted an analytical approach previously demonstrated for finding associations between gene expression and nearby SV breakpoints (9–11). For each of a set of specified genomic region windows in relation to genes, we constructed a somatic SNV matrix by annotating for every sample the presence or absence (using ‘1’ or ‘0’, respectively) of at least one SNV within the given region. For the set of SNVs associated with a given gene within a specified region in relation to the gene (20 kb upstream, 2 kb upstream, 1 kb upstream, gene intron, 1 kb downstream, 3′ UTR and 5′ UTR, with the 20/2/1 kb upstream regions overlapping each other), we assessed the correlation between expression of the gene and the presence of at least one SNV using a linear regression model (with log-transformed expression values). Linear regression models corrected for specific covariates including sample cancer type (as denoted by TCGA/ICGC project for PCAWG and by the above pediatric brain tumor histological types for CBTTC), gender, total mutation burden (i.e. log2 of the total number of SNVs for each tumor profiles, using Mutect2 calls for CBTTC) and gene-level SV breakpoint pattern. For somatic SV breakpoint pattern, we had previously (10) tabulated the relative distances of the somatic SV breakpoint closest to the start of each gene, with a gene X sample relative breakpoint distance matrix being assembled (with maximum distance of 1 Mb imputed if no SV breakpoints found). For these linear regression models, we considered genes with at least three samples associated with an SNV within the given region. The method of Storey and Tibshirani (16) was used to estimate false discovery rates (FDRs) for significant genes. In downstream analyses, we explored the set of genes for which SNVs were significantly associated with expression, after correcting for the above covariates (FDR < 10%) and for which the association was not attributed to gene copy levels (*P* < 0.05, linear model with above covariates plus gene copy).

### Transcription factor (TF) binding site associations

Transcription factor (TF) binding site locations, as determined by ENCODE consortium (17), were obtained from Ensembl (GRCh37/hg19 build for PCAWG data, GRCh38/hg38 build for CBTTC). We used TF sites as identified in the HeLa-S3 cell line (accessed 17 April 2020). To define the set of TF repressors of interest, we selected from the set of TFs with available binding site data those which had Gene Ontology annotation of ‘negative regulation of transcription, DNA-templated’ but not an annotation of ‘activating TF binding.’ By the above criteria, we included 20 TFs in the analysis: BAF170, BRG1, CEBPB, CTCF, E2F1, E2F6, ELK4, EZH2, GABPA, IRF3, JUND, NRSF, PRDM1, REST, RFX5, SMARCA4, SREBF2, STAT3, TCF7L2 and ZHX1. For a set of SNVs of interest (e.g. SNVs 1 or 2 kb upstream of a globally significant gene in a sample with elevated expression of that gene, defined as >0.4 SD from the sample median), the subset of SNVs falling within a TF binding site was annotated, based on the above binding data from ENCODE.

For both PCAWG and CBTTC cohorts, we also examined the SNVs in the 1 or 2 kb upstream region associated with gene over-expression using the BayesPI-BAR2 package (18). BayesPI-BAR2 provides a pipeline for identifying functional non-coding somatic SNVs in cancer patient cohorts, by integrating diverse information such as the spatial distribution of the mutations and a biophysical model for estimating protein binding affinity. We used the default parameters calculating the mutation regulatory blocks, with the minimum number of patients/tumors in a hot mutation region equal to five, the minimum number of SNVs in a hot mutation region equal to ten, and the maximum distance between SNVs in a hot mutation region equal to 30. For the top results, we selected the significant position weight matrices (pwms) at *P* < 0.001 by Bonferroni correction.

### Integrative analyses between SNVs and DNA methylation

TCGA had generated DNA methylation profiles using the Illumina Infinium HumanMethylation450 (HM450) BeadChip array platform (Illumina, San Diego, CA, USA), as previously described (19). Patterns of association of altered DNA methylation with nearby SSV breakpoint focused on the 111 203 array probes falling within CGIs that did not involve X or Y chromosomes (these chromosomes not being included as these would be present or not present or differentially methylated according to patient gender). The analytical approach involving expression data, as described above, was applied similarly to the DNA methylation data. The gene X sample SNV matrices, as constructed above, were joined to the DNA methylation data matrix, in terms of the genes associated with CGIs. We assessed the correlation between methylation of each CGI and the presence of an SSV breakpoint in relation to the CGI-associated gene, using linear regression models (with logit-transformed DNA methylation β values). As with the gene expression analyses, we incorporated specific covariates of relevance into the linear modeling, with FDR (16) used to estimate significant genes. DNA methylation values were logit-transformed to be more aligned with linear model assumptions (20). In downstream analyses, we explored the set of CGI probes for which SNVs were significantly associated with expression (FDR < 10%)—after correcting for sample cancer type, gender, total mutation burden and gene-level SV breakpoint pattern—and for which the association was not attributed to gene copy levels (*P* < 0.05, linear model with above covariates plus gene copy).

### Statistical analysis

All *P*-values were two-sided unless otherwise specified. We utilized linear regression models to associate the expression or methylation of genes with nearby SNVs after adjusting for specific covariates, as described above. In all of the linear models performed in this study, we applied appropriate data transformations to make the data align better with the model assumptions (e.g. log2-transformation for gene expression and logit-transformation for DNA methylation). One-sided Fisher's exact tests or chi-squared tests determined the significance of overlap between two given feature lists. The method of Storey and Tibshirani (16) estimated FDR for significant genes. Visualization using heat maps was performed using JavaTreeview (21) and matrix2png (version 1.2.1) (22).

## RESULTS

### Associations of somatic non-coding SNVs with altered gene expression in adult cancers

Using the PCAWG datasets, we carried out a systematic, pan-cancer analysis of all coding genes, for patterns of expression impacted by nearby somatic SNVs falling within non-coding regions. We aimed to identify genes for which the nearby presence of an SNV significantly associated with changes in expression (based on an analysis of 1220 cases with both WGS and RNA-seq data available, Data S1). We considered non-coding SNVs within fixed genomic regions in relation to each gene. Specifically, we considered somatic SNVs occurring within 20kb upstream of the gene, 2 kb upstream, 1 kb upstream, the gene introns, 1 kb downstream, the gene 3′ Untranslated Region (UTR), and the gene 5′ UTR (the 20, 2 and 1 kb upstream regions overlapping each other). For each of the above regions, we assessed each gene for correlation between one or more SNVs occurring within the region and altered expression. Using linear models, specific variables that may influence expression or SNV patterns—cancer type, gender, total mutation burden, gene-level SV breakpoint pattern (10) and gene-level CNA—were incorporated to determine SNV-expression associations rising above the other variables.

**Figure 1.**
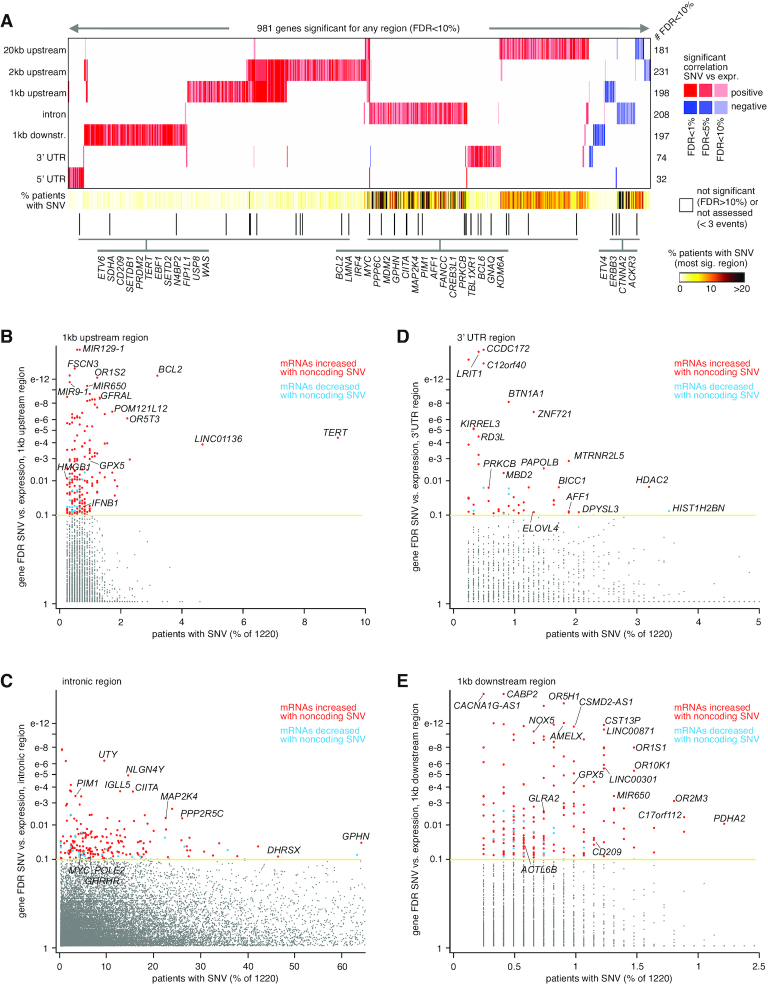
Genes with altered expression associated with nearby somatic SNVs in the PCAWG cohort. (**A**) Heat map of significance patterns, for 981 genes with nearby non-coding SNVs associated with altered expression. For each of several specified genomic region windows in relation to genes (20 kb upstream, 2 kb upstream, 1 kb upstream, gene intron, 1 kb downstream, 3′ UTR and 5′ UTR), the numbers of significant genes (FDR < 10%, with association independent of gene-level CNA) are indicated, based on analysis of the PCAWG cohort (1220 patients). Genes tested for the given region had at least three cases with an SNV in that region. Red, significant positive correlation; blue, significant negative correlation. Genes listed are cancer-related according to COSMIC (38). (**B**) Significance of genes with somatic SNVs for the gene 1 kb upstream region, as plotted (*y*-axis) versus the percent of cases with somatic SNVs. (**C**) Similar to panel (B), but for somatic SNVs in gene intronic regions. (**D**) Similar to panel (B), but for somatic SNVs in gene 3′ UTR regions. (**E**) Similar to part b, but for somatic SNVs in gene 1 kb downstream regions. FDR values are based on linear modeling correcting for sample cancer type, gender, total mutation burden and gene-level SV breakpoint pattern. See also Supplementary Figure S1, Data S1 and S2.

For each of the genomic regions relative to genes that we considered, we found widespread associations between the presence of non-coding SNVs and altered expression, after correcting for covariates (Figure 1A; Supplementary Figure S1a-b and Data S2). For 20 kb upstream, 2 kb upstream, 1 kb upstream, introns, 1 kb downstream, 3′ UTR and 5′ UTR, the numbers of significant genes at FDR < 10% (16) (with association independent of gene-level CNA, see ‘Materials and Methods’ section) were 181, 231, 198, 208, 197, 74 and 32, respectively. For each of these gene sets, more genes positively correlated with SNV event (i.e. expression was higher when at least one SNV was present) than negatively correlated. While the 1 kb upstream and 2 kb upstream regions shared many significant genes, the significant genes of the other regions did not share much overlap (Figure 1A). Interestingly, there were no highly significant overlaps observed between genes altered in association with SNVs and genes altered in association with SVs in PCAWG cohort (9). For example, just 15 genes overlapped between 584 and 359 genes having increased expression associated (*P* < 0.01, linear modeling), respectively, with SV breakpoints and with SNVs in the 20 kb upstream region (*P* = 0.02, one-sided Fisher's exact test), and 11 genes overlapped between genes increased (*P* < 0.01) with within-gene SV breakpoint and genes increased with intronic SNVs (*P* = 0.29, one-sided Fisher's exact).

**Figure 2.**
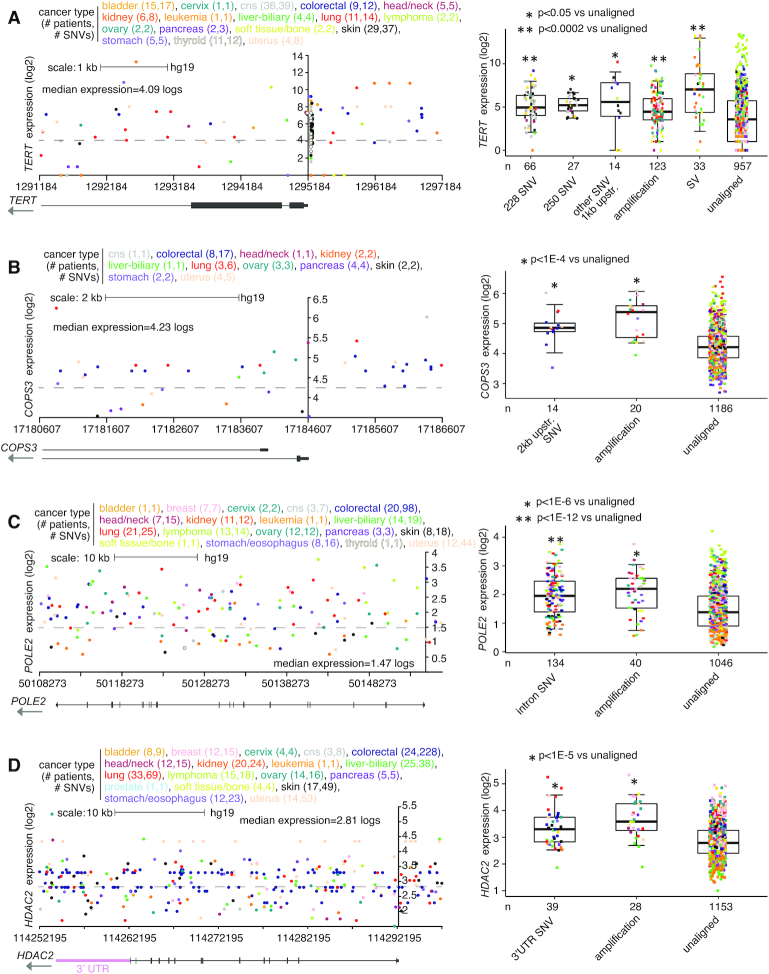
Somatic SNVs associated with increased expression of *TERT*, *COPS3*, *POLE2* and *HDAC2* in PCAWG cohort. (**A**) Left, gene expression levels of *TERT*, corresponding to somatic SNVs located in the genomic region surrounding the gene, including the region 2 kb upstream. Plot represents 145 patients (out of 1220 patients in PCAWG) and 172 SNVs. Each data point represents a single SNV. Multiple SNVs from the same patient will have the same level of expression. Right, box plot of expression for *TERT* by alteration class (‘228 SNV’ and ‘250 SNV,’ known activating promoter mutations (5); ‘other SNV 1 kb upstream.,’ other somatic SNVs 1 kb upstream; ‘amplification,’ approximating copy levels more than 2× greater than that of wild-type; ‘SV,’ somatic SV breakpoint within 100 kb upstream; ‘unaligned,’ cases not involved in any of the above categories). (**B**) Similar to panel (A), but for *COPS3*. Expression plot by genomic position (left) includes the region 2 kb upstream of the gene. Expression plot represents 31 patients and 44 SNVs. Boxplot (right) evaluates differential *COPS3* expression for cases with SNVs 2 kb upstream. (**C**) Similar to panel (A), but for *POLE2*. Expression plot by genomic position (left) includes the region surrounding the entire gene. Expression plot represents 145 patients and 296 SNVs. Boxplot (right) evaluates differential *POLE2* expression for cases with intronic SNVs. (**D**) Similar to panel (A), but for *HDAC2*. Expression plot by genomic position (left) includes the region surrounding the entire gene, including its 3′ UTR. Expression plot represents 224 patients and 580 SNVs. Boxplot (right) evaluates differential *HDAC2* expression for cases with SNVs in 3′ UTR of the gene. *P*-values by *t*-test on log-transformed data. Box plots represent 5, 25, 50, 75 and 95%. For the plots on the left, the *y*-axis is positioned at the gene start.

Perhaps not surprisingly, genomic regions relative to genes representing a larger size—namely, introns and the upstream 20 kb region—tended to involve a higher percentage of patients with SNVs involving the top significant genes (Figures 1B–E and Supplementary Figures S1c and d). Genes significant for the 1 kb upstream region included *TERT*, with SNVs found in 111 PCAWG patients (9%), of which 53 showed elevated expression (>0.4 SD from the sample median) of the gene (Figures 1B and 2A). In addition to the canonical C228T and C250T SNVs previously associated with the upregulation of *TERT* (5,6), additional SNVs 1 kb upstream of *TERT* (involving 14 patients) were also associated with elevated expression but were not recurrent at a specific location (Figure 2A). The significant 1 kb upstream genes included *LINC01136* (SNVs in 4.7% of patients) and *BCL2* (3.2%), with the remaining significant genes representing <2.5% of patients. Similarly, genes significant for the 2 kb upstream, other than the above-noted genes, involved <4% of cases (Supplementary Figure S1c). Genes significantly associated with over-expression for 2 kb upstream region included *COPS3*, an oncogene and a subunit of the COP9 signalosome (23), with SNVs found in 14 patients (Figure 2B). Close to half of the significant genes for intron SNVs each involved more than 10% of patients (Figure 1C), including DNA polymerase epsilon 2, accessory subunit *POLE2*, a gene for which knockdown has shown anti-tumor activity *in vitro* (24), with intronic SNVs involving 155 patients (Figure 2C). The 3' UTR often contains regulatory regions that post-transcriptionally influence gene expression. Genes upregulated in association with SNVs in their 3′ UTR (Figure 1D) included *HDAC2* (Figure 2D), which regulates chromatin plasticity and is frequently deregulated in cancer (25).

**Figure 3.**
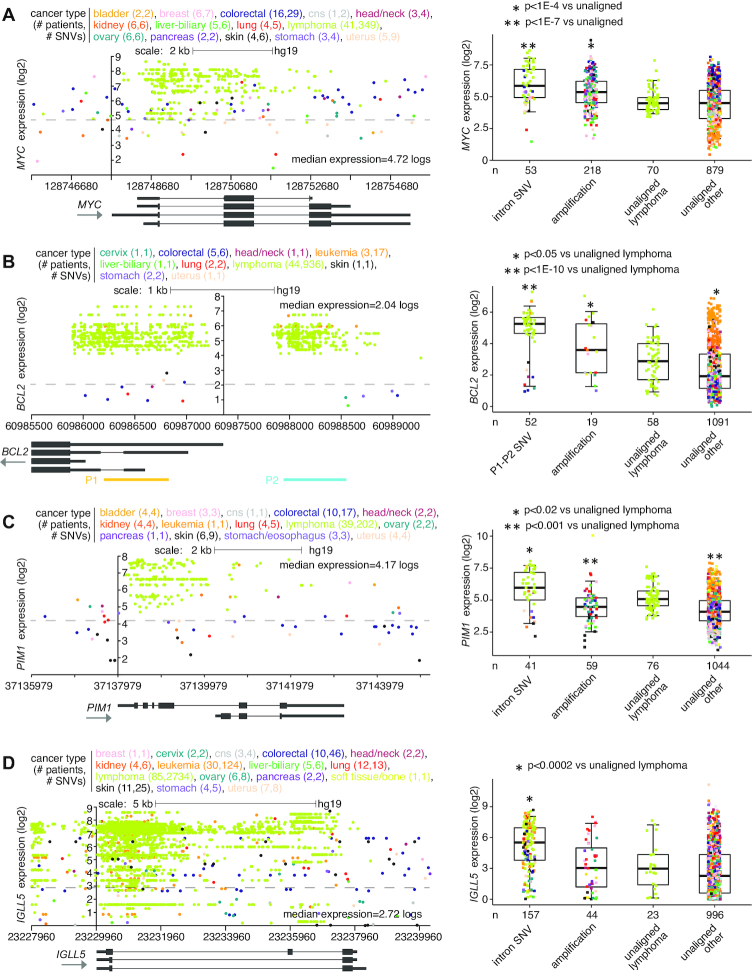
Somatic SNVs associated with increased expression of *MYC*, *BCL2*, *PIM1*, and *IGLL5* in PCAWG lymphoma cases. (**A**) Left, gene expression levels of *MYC*, corresponding to somatic SNVs located in the genomic region surrounding the gene. Plot represents 104 patients (out of 1220 patients in PCAWG) and 437 SNVs (349 in lymphoma cases). Each data point represents a single SNV. Multiple SNVs from the same patient will have the same level of expression. Right, box plot of expression for *MYC* by alteration class (including cases with somatic SNV within introns). (**B**) Similar to panel (A), but for *BCL2*. Expression plot by genomic position (left) includes the P1 and P2 promoter regions. Expression plot represents 61 patients and 968 SNVs (936 in lymphoma cases). Boxplot (right) evaluates differential *BCL2* expression for cases with SNVs in P1 or P2 regions. (**C**) Similar to panel (A), but for *PIM1*. Expression plot by genomic position (left) includes the region surrounding the entire gene. Expression plot represents 84 patients and 358 SNVs (202 in lymphoma cases). Boxplot (right) evaluates differential *PIM1* expression for cases with intronic SNVs. (**D**) Similar to panel (A), but for *IGLL5*. Expression plot by genomic position (left) includes the region surrounding the entire gene. Expression plot represents 187 patients and 2989 SNVs. Boxplot (right) evaluates differential *IGLL5* expression for cases with intronic SNVs. *P*-values by *t*-test on log-transformed data. Box plots represent 5, 25, 50, 75 and 95%. For the plots on the left, the *y*-axis is positioned at the gene start.

For malignant lymphoma cases (PCAWG project MALY-DE), specific genes showed high SNV clustering patterns in proximity to the gene associated with elevated expression (Figure 3). Previous genomic studies of Chronic Lymphocytic Leukemia (CLL) using mutational signatures have demonstrated the activities of activation-induced cytidine deaminase (AID) to underlie such SNV patterns (26). For example, somatic SNV clustering within intron I of *MYC* has long associated with its upregulation in lymphoma (27). This phenomenon is reflected in the PCAWG cohort, involving 41 lymphoma cases and 349 SNVs (Figure 3A). A recent study by Batmanov *et al.* (28) identified SNV clustering near *BCL2* with associated over-expression in follicular lymphoma. A similar phenomenon was observed here in the PCAWG lymphoma cohort, with SNV clustering at both promoter one (P1) and promoter two (P2) of *BCL2*, involving 44 lymphoma cases and 936 SNVs (Figure 3B). *BCL6*, another gene found with SNV clustering in the Batmanov *et al.* study, was also significant in our results set for the 20 kb upstream region (Figure 1A and Supplementary Figure S1d). Other genes with SNV clustering-associated over-expression in PCAWG lymphomas included *PIM1* (29) and *IGLL5* (Figures 3C and D). We also observed SNV clustering for some cases of CLL in PCAWG involving *BCL2* and *IGLL5*. *MYC*, *BCL2*, *PIM1* and *IGLL5* were all found to have SNV hotspots in the non-coding somatic driver analysis study led by PCAWG consortium (2). However, as the SNV patterns were AID-associated and expected to occur later in tumor evolution (26), these genes were not put forth by PCAWG consortium in their final results of somatic non-coding events likely to represent early cancer drivers.

### Associations of somatic non-coding SNVs with altered gene expression in pediatric brain tumors

**Figure 4.**
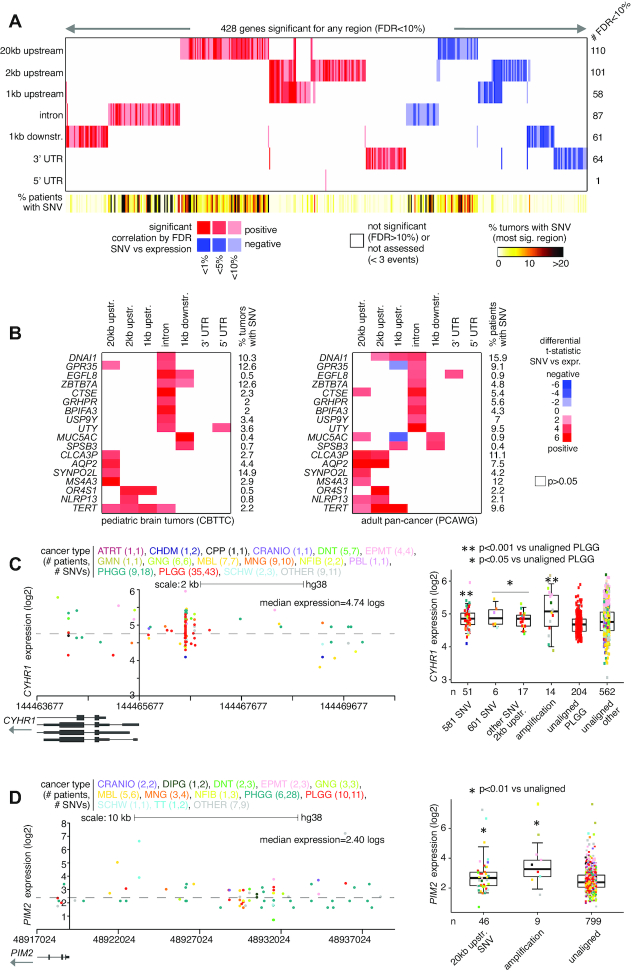
Genes with altered expression associated with nearby somatic SNVs in the CBTTC cohort. (**A**) Heat map of significance patterns, for 428 genes with nearby non-coding SNV associated with altered expression. For each of several specified genomic region windows in relation to genes (20 kb upstream, 2 kb upstream, 1 kb upstream, gene intron, 1 kb downstream, 3′ UTR and 5′ UTR), the numbers of significant genes (FDR < 10%%, with association independent of gene-level CNA) are indicated, based on analysis of the CBTTC pediatric brain tumor cohort (854 tumor samples from 759 patients). Genes tested for the given region had at least three tumors with an SNV in that region. Red, significant positive correlation; blue, significant negative correlation. (**B**) Heat maps representing the set of 18 genes positively correlated (FDR < 25%) with SNV in CTBBC pediatric brain cohort and the genes positively correlated (FDR < 25%) within the PCAWG cohort, for the same regions examined. Red, positive correlation between expression and SNV; blue, negative correlation; white, *P*> 0.05, linear model correcting for sample cancer type, gender, total mutation burden, gene-level SV breakpoint pattern and gene-level CNA. (**C**) Left, gene expression levels of *CYHR1*, corresponding to somatic SNVs located in the genomic region surrounding the gene, including the region 2 kb upstream. Plot represents 94 tumors (out of 854 in CBTTC) and 118 SNVs. Each data point represents a single SNV. Multiple SNVs from the same tumor will have the same level of expression. Right, box plot of expression for *CYHR1* by alteration class, including tumors with SNV 2 kb upstream. PLGG, pediatric low-grade glioma. See ‘Materials and Methods’ section for other pediatric brain tumor cancer type abbreviations. (**D**) Similar to panel (A), but for *PIM2*. Expression plot by genomic position (left) includes the region 20 kb upstream of the gene. Expression plot represents 77 tumors and 44 SNVs. Boxplot (right) evaluates differential *PIM2* expression for cases with SNVs 20 kb upstream. *P*-values by *t*-test on log-transformed data. Box plots represent 5, 25, 50, 75 and 95%. See also Supplementary Figure S2 and Data S3.

As compared to adult cancers, different genomic loci and associated genes are likely to be targeted in the pediatric tumor setting. The above PCAWG expression datasets are primarily representative of adult cancer and did not include any pediatric brain tumor cases in particular. Using the CBTTC datasets (representing 854 tumors from 759 patients), we applied the same analytical approach as applied above to PCAWG datasets, to identify SNV-expression associations for any coding genes. For each of the genomic regions relative to genes that we considered, we found widespread associations between the presence of non-coding SNVs and altered expression, after correcting for covariates (Figure 4A; Supplementary Figures S2a–f and Data S3). For 20 kb upstream, 2 kb upstream, 1 kb upstream, introns, 1 kb downstream, 3′ UTR and 5′ UTR, the numbers of significant genes at FDR < 10% (with association independent of gene-level CNA) were 110, 101, 58, 87, 61, 64 and 1, respectively. We found fewer significant genes for the CBTTC cohort as compared to the PCAWG cohort, likely due in part to the reduced power offered by smaller numbers of tumor profiles in CBTTC, with biological distinctions between pediatric brain tumors and other tumor types probably involved as well.

The set of significant genes for the CBTTC pediatric brain tumor cohort was almost entirely distinct from that of the PCAWG adult pan-cancer cohort, reflecting the unique disease entities the respective cohorts represented. Using more relaxed statistical cutoffs (FDR < 25%) to define significant genes for each cohort, a set of 18 genes positively correlated with SNVs for the same regions examined in both cohorts (Figure 4B). These 18 genes did not represent a highly significant overlap, although *TERT* was significant (*P* < 0.001, linear model) for the 1 kb upstream region in both cohorts. Genes significant for the 2 kb upstream region in the CBTTC cohort included *CYHR1*, with SNVs found in 74 tumors (9%), of which 32 showed elevated expression (>0.4 SD from the median) of the gene (Figure 4C). Recently, *CYHR1* was found to represent both a prognostic marker and a therapeutic target in esophageal squamous cell carcinoma (30). In the CBTTC datasets, *CYHR1* showed SNV hotspots upstream of the gene (Figure 4C), analogous to SNV patterns involving *TERT* upregulation. Most tumors with an upstream SNV were pediatric low-grade gliomas. Two somatic SNV hotspots in the *CYHR1* promoter region were A→C at position 581 (hg38 coordinates), involving 51 tumors and T→G at position 601, involving nine tumors (three of which also had A581C). Five tumors also had A→C at position 757. Most of the other significant genes did not show the sort of tight SNV clustering patterns observed for *CYHR1*. Instead, SNV-expression associations would involve SNVs positioned throughout the region. One example involved the oncogene *PIM2*, a gene typically associated with leukemias (31), for which SNVs across the region 20kb upstream of the gene associated with elevated expression in pediatric brain tumors (Figure 4D).

### SNV densities and transcription factor (TF) binding sites involving SNV-associated altered expression

**Figure 5.**
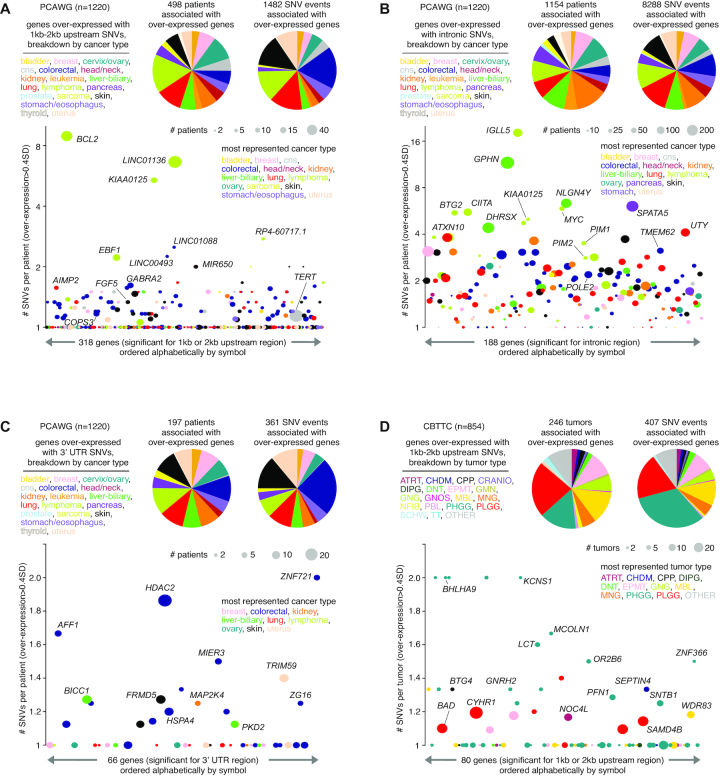
Breakdowns by cancer type and SNV density for genes with altered expression associated with nearby somatic SNVs. (**A**) Based on the set of 318 genes significant for either 1 or 2 kb upstream region in PCAWG cohort (from Figure 1A), pie charts provide breakdowns by cancer type according to the following: (1) the 498 patients for which an SNV in the region was associated with elevated expression of a gene (expression > 0.4SD from median for the case harboring the breakpoint) and (2) the 1482 gene–SNV associations involving over-expression. For the 1482 gene–SNV associations, just one SNV for each patient is considered in the instance of multiple SNVs. Scatterplot below orders the 318 significant genes alphabetically by name, plotting on the *y*-axis the average number of SNVs per patient involving each gene. Data points are sized according to the total number of patients with both SNV and elevated expression, and data points are colored according to the most represented cancer type. (**B**) Similar to panel (A), but for the set of 188 genes significant for the intronic region in PCAWG cohort. (**C**) Similar to panel (A), but for the set of 66 genes significant for the 3′ UTR region in PCAWG cohort. (**D**) Similar to panel (A), but for the set of 80 genes significant for either 1 or 2 kb upstream region in CBTTC cohort. See ‘Materials and Methods’ section for pediatric brain tumor cancer type abbreviations. See also Supplementary Figure S3, Data S4 and S5.

In PCAWG and CBTTC cohorts, significant SNV–gene associations involved almost all cancer types represented (Figure 5A–D; Supplementary Figures S3a-b and Data S4 and S5). For some genes, such as those above associated primarily with PCAWG lymphoma cases (*MYC*, *BCL2*, *PIM1* and *IGLL5*, Figure 3), there was a very high clustering of SNVs in the region relative to the gene examined. Such patterns can represent regulatory mutation blocks that affect the binding affinity of TFs (28). We quantified this SNV clustering for each gene significant for any of the following regions: 1–2 kb upstream (considering genes arising from either the 1 or 2 kb region), intronic and 3′ UTR. For each significant gene for the given region, we calculated the average number of SNVs per patient, for SNVs involving patients with elevated expression of the gene (>0.4 SD from the median). For the 1–2 kb upstream region, significant genes in the PCAWG cohort with a very high density of SNVs included *BCL2* as mentioned above and long non-coding RNA *LINC01136*, primarily involving lymphoma cases (Figure 5A). In contrast, *TERT*-associated SNVs involved ∼57 patients but without a high number of SNVs per patient. For the gene intronic region, significant genes in the PCAWG cohort tended overall to have higher numbers of SNVs per patient as compared to that of the other regions, in part due to the larger region represented by introns (Figure 5B), with notable genes including *IGLL5* (most represented in lymphoma cases) and *GPHN* (most represented in liver cancer). On the other hand, genes significant for the 3′ UTR region for the PCAWG cohort (Figure 5C) and genes significant for the various regions examined in the CBTTC cohort (Figures 5D; Supplementary Figure S3a and b) both tended to have lower numbers of SNVs per patient.

**Figure 6.**
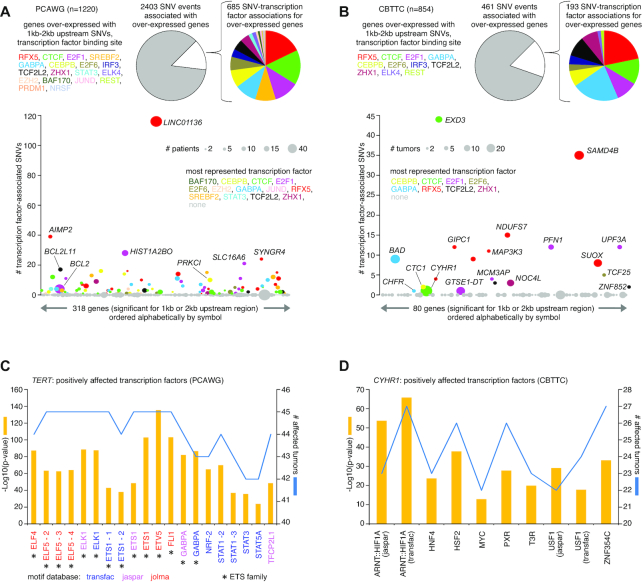
Breakdowns by impacted TF binding site for genes with altered expression associated with nearby somatic SNVs. (**A**) Based on the set of 318 genes significant for either 1 or 2 kb upstream region in PCAWG cohort (from Figure 1A), pie charts provide breakdowns by impacted TF binding set according to the 2403 gene–SNV associations involving over-expression (expression > 0.4 SD from median for the case harboring the breakpoint). For the 2403 gene–SNV associations, multiple SNVs for each patient may be considered. Scatterplot below orders the 318 significant genes alphabetically by name, plotting on the *y*-axis the total number of TF-associated SNVs involving over-expression. Data points are sized according to the total number of patients with both SNV and elevated expression, and data points are colored according to the most represented TF. (**B**) Similar to panel (A), but for the set of 80 genes significant for either 1 or 2 kb upstream region in CBTTC cohort. (**C**) For *TERT*, the set of TFs predicted to be positively affected by SNVs associated with gene over-expression in the PCAWG cohort, based on the BayesPI-BAR2 package (18). *P*-values based on Wilcoxon rank-sum test after Bonferroni correction. Some TFs are represented by multiple Position Weight Matrix models, their instances indicated by a number or the motif source. (**D**) Similar to panel (A), but for *CHYR1* in the CBTTC cohort.

Somatic SNVs could conceivably disrupt TF binding, including TFs with repressor functions. From ENCODE consortium (17), we obtained binding site locations for a set of 20 TF repressors, to determine which of the SNVs associated with gene over-expression may fall within these binding sites. For the PCAWG cohort, of the 2403 SNV events in the 1–2 kb upstream region associated with over-expressed genes (>0.4 SD from the median for the given sample), 329 (13.7%) involved TF repressor binding, representing 685 SNV–TF associations with some SNVs overlapping with more than one TF (Figure 6A). The most represented TFs in this SNV set included RFX5 (122 SNVs, some involving other TFs), CTCF (110), E2F1 (78), GABPA (69), SREBF2 (65) and CEBPB (55). For the CBTTC cohort, of the 461 SNV events in the 1–2 kb upstream region associated with over-expressed genes, 80 (17.4%) involved TF repressor binding (Figure 6B), representing 193 SNV-TF associations. The most represented TFs in this SNV set included GABPA (42 SNVs), RFX5 (42), CTCF (23), E2F1 (19), TCF7L2 (18), ZHX1 (15), and CEBPB (14).

Somatic SNVs could also conceivably create new TF binding sites. We used the BayesPI-BAR2 algorithm (18) to evaluate possible TF binding effects represented by the clustered SNV patterns associated with over-expressed genes. For the PCAWG cohort, of the 2403 SNV events in the 1–2 kb upstream region associated with over-expressed genes, just four genes—*TERT*, *BCL2*, *KIAA0125* and *LINC01136*—involved tight clusters of at least 10 SNVs within 30 bases of each other (‘Materials and Methods’ section). Of these genes, *TERT* SNVs were associated with predicted increased binding of ETS family TFs (Figure 6C and Supplementary Data S4), as expected. *BCL2* SNVs were associated with disruption of FOX TF family members (Supplementary Data S4), consistent with previous findings outside of PCAWG (28). For the CBTTC cohort, of the 461 SNV events in the 1–2 kb upstream region associated with over-expressed genes, just two genes, *CYHR1* and *GTSE1* divergent transcript (*GTSE1-DT*) involved tight SNV clusters. *CYHR1* SNVs were associated with predicted increased binding of several TFs, including ARNT::HIF1A, HNF4, HSF2, PXR, T3R, USF1 and ZNF354C (Figure 6D and Supplementary Data S5).

Furthermore, we examined the set of SNVs falling within introns and associated with over-expression of the gene, for those which might represent splicing alterations. Using a previously generated catalog of SNVs near exon–intron boundaries in the PCAWG cohort (8), just 40 of the 24 762 intron-related SNV events in our set involved exon–intron boundaries, with only five SNV events associated with a change in splicing. Therefore, the phenomenon represented by the intron SNV-expression associations uncovered by our study would not appear to involve altered splicing.

### Associations of somatic non-coding SNVs with altered methylation of CpG Islands (CGIs)

**Figure 7.**
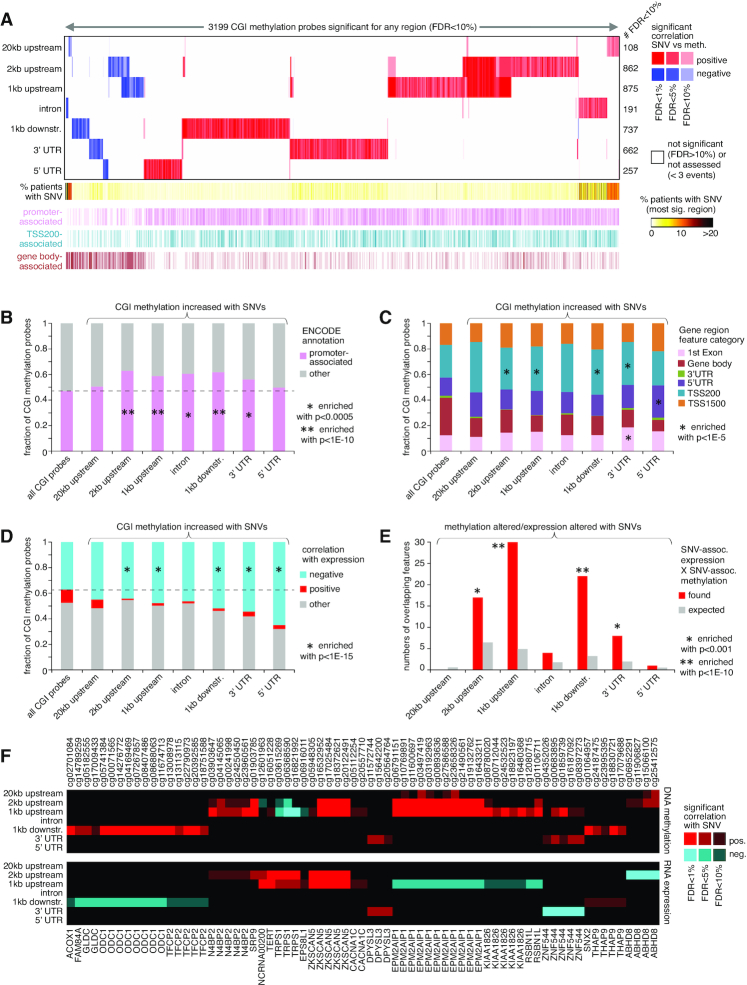
CGIs with altered DNA methylation associated with nearby somatic SNVs in the PCAWG cohort. (**A**) Heat map of significance patterns, for 3199 CGI probes (out of 111 203 on array platform) with nearby non-coding SNV associated with altered DNA methylation. For each of several specified genomic region windows in relation to CGI-associated genes (20 kb upstream, 2 kb upstream, 1 kb upstream, gene intron, 1 kb downstream, 3′ UTR and 5′ UTR), the numbers of significant CGI probes (FDR < 10%, with association independent of gene-level CNA) are indicated, based on analysis of the PCAWG cohort (568 patients). CGI probes tested for the given region had at least three cases with an SNV in that region. Red, significant positive correlation; blue, significant negative correlation. CGI probe annotation is provided along the bottom (e.g. TSS200, probe located within 200 bases from the transcription start site). (**B**) Fraction of promoter-associated CGIs (ENCODE annotation of the 450K platform), for the CGI probes associated with increased methylation (from panel (A)), according to genomic region examined. *P*-values by chi-square test. (**C**) Breakdown by probe position relative to gene, for the CGI probes associated with increased methylation (from panel (A)), according to genomic region examined. *P*-values by chi-square test. (**D**) Breakdown by the overall correlation between expression and DNA methylation across cancer cases, for the CGI probes associated with increased methylation with nearby SNV (from panel (A)), according to genomic region examined. Significant correlation defined as FDR < 5% by linear model, correcting for both cancer type and gene-level CNA (based on 1482 TCGA cases (10)). Enrichment *P*-values by chi-square test. (**E**) Overlaps between CGI probes with SNV-associated altered methylation and nearby genes with corresponding SNV-associated altered expression (FDR < 10% for both feature sets, regardless of direction), according to genomic region examined. Chance expected overlaps between feature sets are also represented. Enrichment *P*-values by one-sided Fisher's exact test. (**F**) The top set of 71 CGI methylation probes (representing 21 genes) significantly associated with nearby SNVs, for which the expression versus SNV association for the corresponding regions were also significant (from panel (E)). The top panel represents DNA methylation associations, and the bottom panel represents gene expression associations. Red, significant positive correlation with SNV; blue, significant negative correlation. See also Supplementary Figure S4 and Data S6.

While non-coding somatic SNVs have been understood to impact the expression of individual genes such as *TERT*, the PCAWG datasets included 568 cases with DNA methylation data. These data presented an opportunity for us to carry out an analogous survey of associations between non-coding somatic SNVs and DNA methylation patterns across these cancers. We joined the gene by sample SNV matrices, as constructed above for analysis of gene expression (Figure 1A), to the DNA methylation data matrix of 568 cases, in terms of the genes associated with CGIs. Using linear regression models, we assessed the correlation between methylation of each CGI and the presence of an SNV in the given region in relation to the CGI-associated gene, with the same covariates used for the expression analysis. With this approach, we examined 111 203 CGI DNA methylation probes, involving 13 043 associated genes, and the above seven genomic regions in relation to genes. For each genomic region, we found hundreds of CGI probes consistently altered in methylation by nearby SNVs (Figure 7A and Supplementary Data S6). For 20 kb upstream, 2 kb upstream, 1 kb upstream, introns, 1 kb downstream, 3′ UTR and 5′ UTR, the numbers of significant genes at FDR < 10% by linear model were 108, 862, 875, 191, 737, 662 and 257, respectively. In contrast to the gene expression results, there were notably fewer associations between methylation and SNVs within the 20 kb upstream and gene intronic regions, even though these two regions were the largest.

More CGI features were positively correlated (i.e. showed increased methylation) with somatic SNV events than were negatively correlated, and positively versus negatively correlated CGI sets showed distinctive characteristics with respect to each other. CGI probes with SNV-associated increased methylation tended to be promoter-associated (Figure 7B) and were enriched for probes within 200 bp of the gene transcriptional start site (Figure 7C). In contrast, CGI probes with SNV-associated decreased methylation were markedly anti-enriched for promoter-associated probes (Figure 7A and Supplementary Figure S4a) but were highly enriched for gene body CGIs (Figure 7A and Supplementary Figure S4b). The above CGI probe location associations for SNV-related methylation increases or decreases were similar to associations involving SV breakpoint-related methylation increases or decreases, respectively (10). When considering the overall correlation between expression and DNA methylation across cancer cases, the CGI probes with SNV-associated increased methylation were highly enriched for genes negatively correlated between methylation and expression (Figure 7D). We did not observe this enrichment pattern for the CGI probes with SNV-associated decreased methylation (Supplementary Figure S4c).

Classical DNA methylation at the CGIs of promoters causes stable silencing of genes (32). We examined the overlap between CGI probes with SNV-associated altered methylation and the related genes with corresponding SNV-associated altered expression (FDR < 10% by linear model for each set). For the 2 kb upstream, 1 kb upstream, 1 kb downstream and 3' UTR regions, there were significant overlaps between CGI probes with SNV-associated altered methylation and nearby genes with corresponding SNV-associated altered expression (Figure 7E). These overlaps altogether represented 71 CGI methylation probes and 21 genes (Figure 7F), most of these expression-methylation correlations being in the inverse direction. At the same time, the 71 CGI probes represented just a small fraction of the 3199 globally significant probes (from Figure 7A), of which 1702 were significantly correlated between methylation and expression (1497 inversely correlated, using FDR < 5% by linear model, correcting for both cancer type and gene-level CNA and based on 1482 TCGA cases (10)). This finding indicates that some genes may not be globally associated with SNV-mediated expression changes across all tumors, but these genes may still be impacted by SNV-association methylation alterations in a subset of tumors, while influenced by additional variables in the other tumors.

## DISCUSSION

This study provides a comprehensive catalog of mRNAs appearing deregulated by nearby non-coding somatic SNVs, across both adult cancers of various types and pediatric brain tumors of various histologies. The finding of the PCAWG adult cancer cohort yielding different sets of top significant genes from those of the CBTTC pediatric brain tumor cohort would underscore the notion that pediatric cancers represent a markedly different set of diseases from adult cancers (33). Other factors that may be involved in observed discrepancies between PCAWG and CBTTC results include the smaller CBTTC cohort, younger patients failing to accumulate a significant number of mutations, and lack of representation of similar pediatric cancers in the adult patient cohort. By using gene expression as a filter for assigning significance to SNVs, our analytical approach is not limited by our incomplete understanding of the functional role of the non-coding genome. Also, SNV patterns that are not highly recurrent (but falling across a given region) may contribute to expression associations. Non-coding somatic SNVs can potentially create new TF binding sites for transcriptional activators or disrupt existing binding sites for transcriptional repressors. Our study observed an overall trend of non-coding SNVs resulting in more upregulation versus downregulation of nearby genes. In many instances, it may be challenging to determine the precise mechanism for SNV-mediated deregulation. We based our determination of gene-level significance on a fixed genomic region window relative to the gene. However, every gene would have its unique regulatory landscape, and a modified region window might have better revealed an association for some genes. For many of our top genes, a functional role in cancer may not yet be established. Also, in a few instances, an upregulated gene may represent a tumor suppressor. One challenge with interpreting our results would be to understand which of the deregulated genes are genuinely contributing to the disease, besides the genes with well-established oncogenic roles. In future experimental studies, as more functional data establish previously understudied genes as having potential oncogenic roles, our results would represent a resource to help determine how such genes may be deregulated in the human tumor setting.

In contrast to analytical approaches that focus first on SNV hotspots or regulatory regions or motifs, our approach primarily focuses on associated expression changes, and the interpretations as to the roles of our top significant genes in cancer may differ from that of a conventional ‘drivers’ study. For example, several of our top genes showed a tight SNV clustering pattern associated with elevated expression, including *MYC*, *BCL2*, *PIM1* and *IGLL5* in lymphoma. However, in the PCAWG-led Rheinbay *et al.* drivers study (2), a post-filtering of significant hits was performed to remove those genes involving mutations caused by mutational processes. In particular, Rheinbay *et al.* identified genes with AID-related mutations in lymphoma in the first round of the PCAWG analyses as candidate drivers (due to the clustered mutation patterns). Still, they were not part of the set of driver genes put forth by PCAWG in their final results. The rationale for this is that these AID-related gene alterations would represent subsequent events, which presumably resulted from the somatic alteration of ‘true’ drivers that would have initiated the AID mutation signature processes at an earlier stage of the disease. In our present study, the understanding of our results involving AID-related genes is consistent with that of Rheinbay *et al.* However, while genes such as *BCL2* and *MYC* may not satisfy a classical definition of drivers in terms of their mutation patterns, they nevertheless would play an important role in the disease. In lymphoma, we find that only specific genes appear altered for expression in association with clustered non-coding SNV patterns. These genes include those with well-known oncogenic roles, and for which non-coding SNV associations have previously been reported for lymphoma cohorts outside of PCAWG (27–29). A number of our top genes may well represent passenger SNV events. However, the elevated expression observed for several genes previously determined to have functional roles in cancer seems likely to represent disease contributors, even if such genes may not fit within a dichotomous model of drivers versus passengers (3).

This present study has revealed widespread associations in human cancer of non-coding somatic SNVs with CGI methylation of nearby gene promoters. Specific CGIs appearing recurrently and non-randomly altered in association with nearby SNVs across cancers is intriguing and suggestive of a selection process in the disease. This phenomenon would be distinct from that involving mutation of chromatin modifier genes, which results in a more general, widespread impact on DNA methylation (19). CGIs with SNV-associated methylation increases versus decreases respectively represented different classes of CGI annotations, similar to the CGI classes involved in SV-associated methylation alterations (10). Whether the observed altered DNA methylation patterns would be the driving cause or the result of somatic mutation remains an open question. On the one hand, it has been understood that DNA repair in cancer, particularly involving double-stranded breaks, can lead to altered CpG methylation at the repair site (34–36). On the other hand, over-representation of mutations at CpG dinucleotides has implicated DNA methylation in the generation of oncogenic point mutations, where the DNA methyltransferase enzyme itself might contribute to the high rate of transitions seen at CpG dinucleotides (37). Both of the above could conceivably be at work in our results. Altered DNA methylation in association with SNVs would represent just one potential mechanism for altering gene expression, with many other mechanisms involving different genes and different tumors. There would be no common explanation for all the differential expression and methylation patterns observed in the context of nearby non-coding SNVs, as the regulatory landscape for each gene is unique. As more data from more cancer genomes become available, future studies will continue to refine our knowledge of the set of non-coding somatic alterations that contribute to cancer.

## ETHICS APPROVAL

Cancer molecular profiling data were generated through informed consent as part of previously published studies and analyzed in accordance with each original study's data use guidelines and restrictions.

## Supplementary Material

zcab001_Supplemental_Files

## Data Availability

All data used in this study are publicly available. For PCAWG data, SNV calls, copy number data and expression data are available at the ICGC Data Portal (https://dcc.icgc.org/pcawg). For CBTTC data, molecular data are available through the public project on the Kids First Data Resource Portal and Cavatica (https://cbttc.org/) and the PedCBioPortal (https://pedcbioportal.org/). Source code in R for the linear modeling integrating SNV with expression data, with example data files, is available at Github (https://github.com/chadcreighton/SNV-expression_integration).
