# Supplementary material for: Systematic identification of non-coding somatic single nucleotide variants associated with altered transcription and DNA methylation in adult and pediatric cancers
Source: NAR Cancer. 2021 Feb 1;3(1):zcab001. doi: 10.1093/narcan/zcab001 (PMC7849833; doi:10.1093/narcan/zcab001)
Supplement: zcab001_Supplemental_Files [file zcab001_supplemental_files.zip › Creighton_SNV_expression_Supplementary_info_120420e.docx]

**
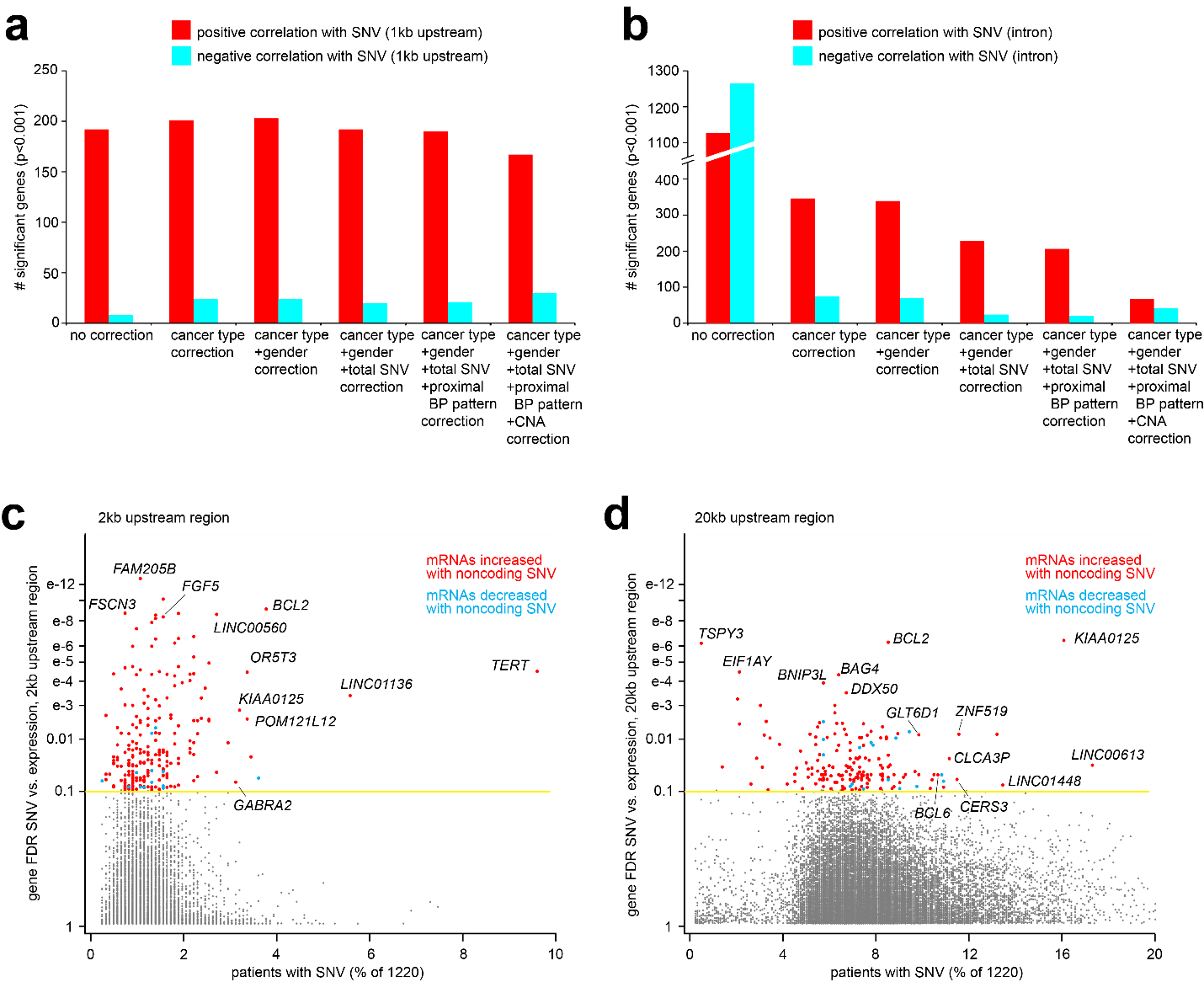
**

**Figure S1, related to Figure 1. Additional information regarding genes with altered expression associated with nearby somatic SNVs in the PCAWG cohort. (a)** Numbers of significant genes (p<0.001 by linear model using log-transformed expression values), showing a correlation between expression and SNVs occurring 1kb upstream of the gene, based on 1220 cases with RNA-seq data. Linear regression models evaluated significant associations when correcting for specific covariates, as indicated. **(b)** Similar to part a, but for SNVs occurring in gene intronic regions. **(c)** Significance of genes with somatic SNVs for the gene 2kb upstream region, as plotted (Y-axis) versus the percent of cases with somatic SNVs. **(d)** Similar to part c, but for somatic SNVs in the gene 20kb upstream region.

**
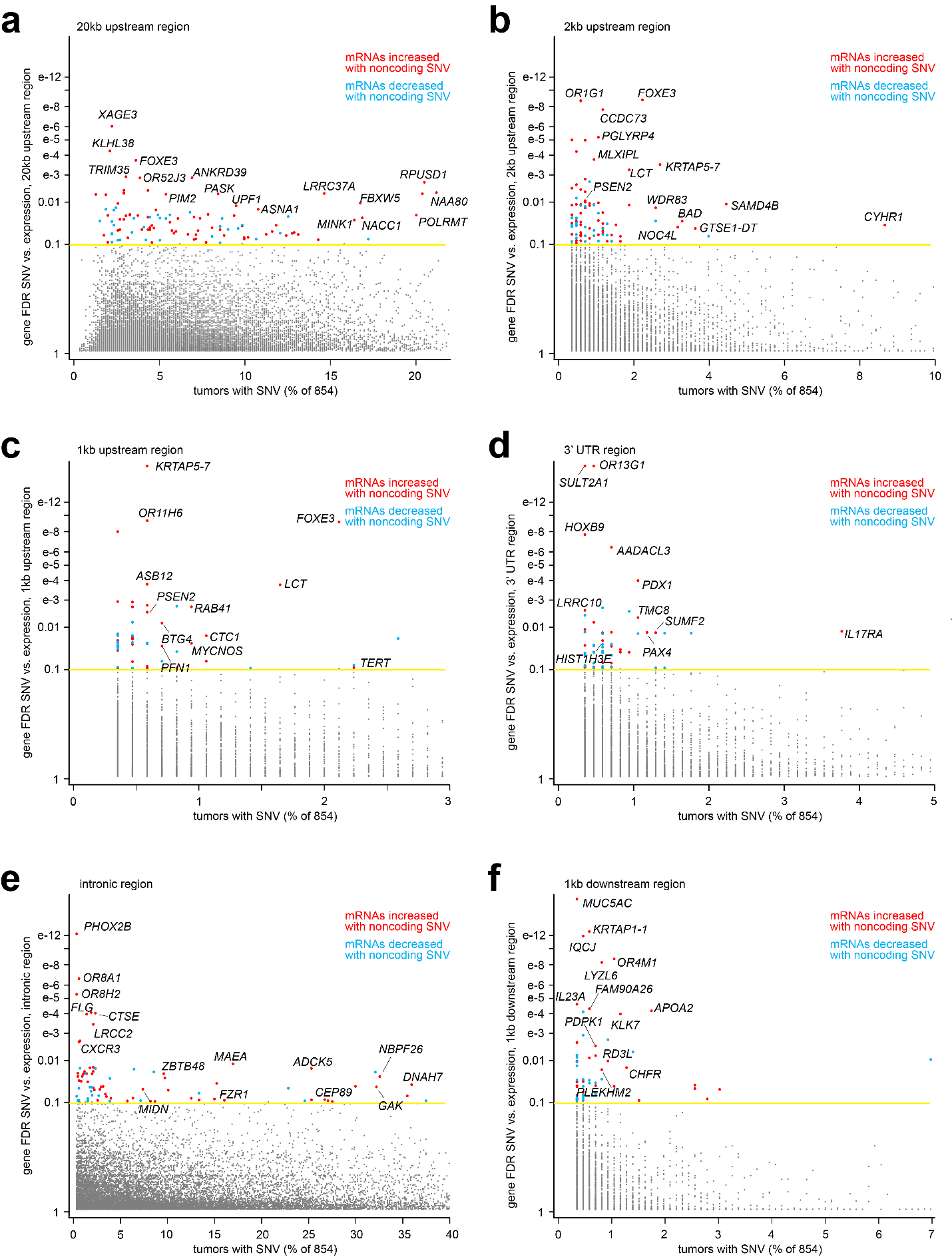
**

**Figure S2, related to Figure 2. Additional information regarding genes with altered expression associated with nearby somatic SNVs in CBTTC cohort. (a)** Significance of genes with somatic SNVs for the gene 20kb upstream region, as plotted (Y-axis) versus the percent of tumors with somatic SNVs. **(b)** Similar to part a, but for somatic SNVs in gene 2kb upstream regions. **(c)** Similar to part a, but for somatic SNVs in gene 1kb upstream regions. **(d)** Similar to part a, but for somatic SNVs in gene 3’UTR regions. **(e)** Similar to part a, but for somatic SNVs in gene intronic regions. **(f)** Similar to part a, but for somatic SNVs in gene 1kb downstream regions. FDR values are based on linear modeling correcting for sample cancer type, gender, total mutation burden, and gene-level SV breakpoint pattern.

**
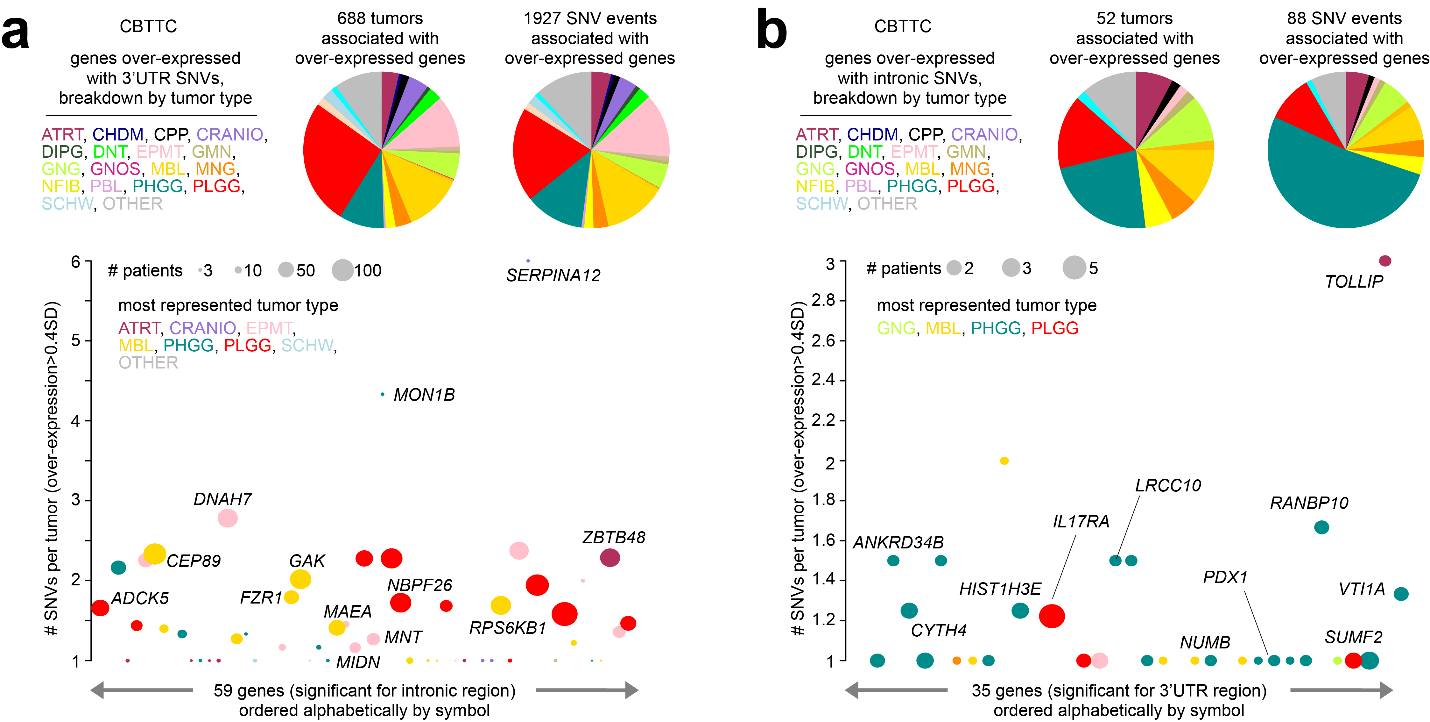
**

**Figure S3, related to Figure 5. Additional breakdowns by cancer type and SNV density for genes with altered expression associated with nearby somatic SNVs. (a)** Based on the set of 59 genes significant (from main Figure 4a) for either 1kb or 2kb upstream region in CBTTC pediatric brain cohort, pie charts provide breakdowns by cancer type according to the following: 1) the 688 patient tumors for which an SNV in the region was associated with elevated expression of a gene (expression>0.4SD from median for the case harboring the breakpoint) and 2) the 1927 gene-SNV associations involving over-expression. For the 1927 gene-SNV associations, just one SNV for each patient is considered in the case of multiple SNVs. Scatterplot below orders the 59 significant genes alphabetically by name, plotting on the y-axis the average number of SNVs per patient involving each gene. Data points are sized according to the total number of patients with both SNV and elevated expression, and data points are colored according to the most represented cancer type. See Methods for pediatric brain tumor cancer type abbreviations. **(b)** Similar to part a, but for the set of 35 genes significant for the 3’UTR region in the CBTTC cohort.

**
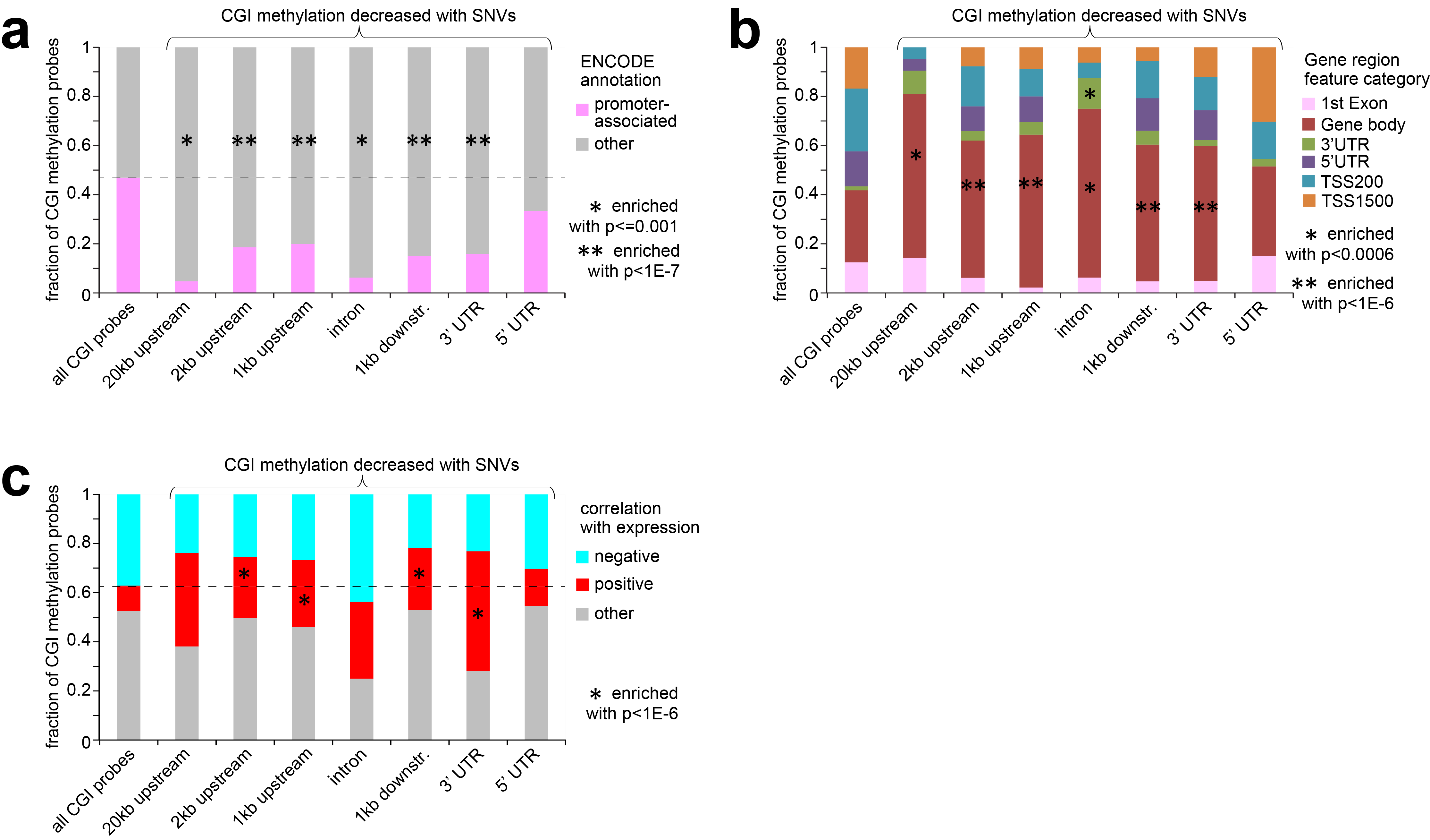
**

**Figure S4, related to Figure 7. Additional information on CGIs with altered DNA methylation associated with nearby somatic SNVs. (a)** Fraction of promoter-associated CGIs, for the CGI probes associated with decreased methylation (from main Figure 7a), according to genomic region examined. P-values by chi-square test. **(b)** Breakdown by probe position relative to gene, for the CGI probes associated with decreased methylation (from main Figure 7a), according to genomic region examined. P-values by chi-square test. **(c)** Breakdown by overall correlation between expression and DNA expression across cancer cases, for the CGI probes associated with decreased methylation (from main Figure 7a), according to genomic region examined. Significant correlation defined as FDR < 5% by linear model, correcting for both cancer type and gene-level CNA (based on 1482 TCGA cases[1]). Enrichment p-values by chi-square test.

References

1. Zhang Y, Yang L, Kucherlapati M, Hadjipanayis A, Pantazi A, Bristow C, Lee E, Mahadeshwar H, Tang J, Zhang J, et al: **Global impact of somatic structural variation on the DNA methylome of human cancers.** *Genome Biol* 2019, **20:**209.
