# Supplementary material for: Systematic identification of non-coding somatic single nucleotide variants associated with altered transcription and DNA methylation in adult and pediatric cancers
Source: NAR Cancer. 2021 Feb 1;3(1):zcab001. doi: 10.1093/narcan/zcab001 (PMC7849833; doi:10.1093/narcan/zcab001)
Supplement: zcab001_Supplemental_Files [file zcab001_supplemental_files.zip › List of Supplementary Data.docx]

**Data S1.** PCAWG and CBTTC cancer cases examined in this study.

**Data S2.** From PCAWG datasets, the complete set of gene-level correlations between expression and nearby SNV event, according to region examined (20kb upstream, 2kb upstream, 1kb upstream, gene intron, 1kb downstream, 3’ UTR, and 5’ UTR). For selected genes (*TERT*, *COPS3*, *POLE2*, *HDAC2*, *MYC*, *BCL2*, *PIM1*, and *IGLL5*), associated SNVs and gene expression values are provided (based on Figures 2 and 3).

**Data S3.** From CBTTC datasets, the complete set of gene-level correlations between expression and nearby SNV event, according to region examined (20kb upstream, 2kb upstream, 1kb upstream, gene intron, 1kb downstream, 3’ UTR, and 5’ UTR). For selected genes (*CYHR1* and *PIM2*), associated SNVs and gene expression values are provided (based on Figure 4).

**Data S4.** From PCAWG datasets, the set of SNVs associated with elevated gene expression (FDR<10% for the gene and >0.4SD from the median for the sample profile) for the following regions in relation to genes: 1-2kb upstream, intron, and 3’ UTR. Also provided are cancer type and transcription factor associations involving the top significant genes (based on Figures 5 and 6), as well as the top TF motif hits by BayesPI-BAR for the 1-2kb upstream SNVs.

**Data S5.** From CBTTC datasets, the set of SNVs associated with elevated gene expression (FDR<10% for the gene and >0.4SD from the median for the sample profile) for the following regions in relation to genes: 1-2kb upstream, intron, and 3’ UTR. Also provided are cancer type and transcription factor associations involving the top significant genes (based on Figures 5 and 6 and S3), as well as the top TF motif hits by BayesPI-BAR for the 1-2kb upstream SNVs.

**Data S6.** Complete set of probe-level CGI correlations between DNA methylation and nearby SNV event, according to region examined (20kb upstream, 2kb upstream, 1kb upstream, gene intron, 1kb downstream, 3’ UTR, and 5’ UTR).
